# Clinical applicability of cystatin C-based renal function assessment and HPLC-based therapeutic drug monitoring for predicting cefepime overexposure

**DOI:** 10.1186/s40780-026-00619-6

**Published:** 2026-08-13

**Authors:** Kyohei Sugiyama, Tomoki Kimura, Nanaho Fukumoto, Madoka Kibe, Yukako Suyama, Kenta Ito, Kazuko Sakurai, Kunihiko Itoh, Tomonobu Uchino

**Affiliations:** 1 Department of Clinical Pharmacology & Genetics, School of Pharmaceutical Sciences, University of Shizuoka, 52-1 Yada Suruga-ku, Shizuoka, 422-8526 Japan; 2 Department of Clinical Pharmacology & Genetics, Graduate School of Pharmaceutical Sciences, University of Shizuoka, 52-1 Yada, Suruga, Shizuoka, 422-8526 Japan; 3 Laboratory of Clinical Pharmacogenomics, Research Support Center, Shizuoka General Hospital, 4-27-1 Kita-Ando, Aoi-ku, Shizuoka, 420-8527 Japan; 4 Department of Pharmacy, Shizuoka General Hospital, 4-27-1 Kita Ando Aoi-ku, Shizuoka, 420-8527 Japan; 5 Department of Clinical Laboratory Medicine, Shizuoka General Hospital, 4-27-1 Kita Ando Aoi-ku, Shizuoka, 420-8527 Japan

**Keywords:** Cefepime, Cystatin C, High-performance liquid chromatography, Neurotoxicity, Renal function assessment

## Abstract

**Background:**

Cefepime (CFPM)-associated neurotoxicity is linked to elevated plasma concentrations, particularly in patients with impaired renal function. Although cystatin C-based estimated glomerular filtration rate (eGFRcys) has been proposed as a potentially superior marker of renal function for dose optimization, its clinical applicability for predicting CFPM exposure remains insufficiently established. We developed a clinically applicable high-performance liquid chromatography (HPLC) method for plasma CFPM quantification and evaluated the clinical utility of eGFRcys, with particular focus on its discordance with creatinine clearance (Ccr).

**Methods:**

This prospective study enrolled 22 hospitalized patients receiving CFPM. Steady-state trough concentrations were measured using the developed HPLC method. Overexposure was defined as ≥ 16 µg/mL, based on a previously reported threshold associated with an increased risk of cefepime neurotoxicity. Associations between trough concentrations and renal function indices were evaluated using Spearman’s correlation, and the impact of discordance between Ccr and body surface area-adjusted eGFRcys [eGFRcys(BSA)] on CFPM exposure was investigated.

**Results:**

The HPLC assay demonstrated excellent analytical performance, including high precision and robust chromatographic separation under polypharmacy conditions, enabling reliable quantification in routine clinical practice. Despite Ccr-guided dosing, 55% of patients had trough concentrations ≥ 16 µg/mL. Renal function-normalized dose correlated with trough concentrations, with the strongest association observed for eGFRcys(BSA) (ρ = 0.86, *P* < 0.001). Notably, all patients with preserved renal function according to Ccr (≥ 60 mL/min) but impaired renal function according to eGFRcys(BSA) (< 60 mL/min) exhibited trough concentrations ≥ 16 µg/mL, suggesting that Ccr-based assessment alone may underestimate the risk of CFPM overexposure. Furthermore, all patients with impaired renal function (Ccr < 60 mL/min) also exhibited trough concentrations ≥ 16 µg/mL.

**Conclusions:**

This validated HPLC method provides a practical approach for routine CFPM therapeutic drug monitoring using conventional laboratory infrastructure. eGFRcys(BSA) showed the strongest correlation with CFPM exposure among the renal function indices evaluated and may improve identification of patients at risk of overexposure. However, renal function assessment alone was insufficient to explain exposure variability in patients with impaired renal function. These findings support the clinical applicability of eGFRcys(BSA) for CFPM dose optimization while highlighting the complementary role of HPLC-based therapeutic drug monitoring for individualized minimize the risk of overexposure.

**Supplementary Information:**

The online version contains supplementary material available at https://doi.org/10.1186/s40780-026-00619-6.

## Background

Cefepime (CFPM), a fourth-generation cephalosporin, is widely used to treat severe infections owing to its broad antimicrobial spectrum. It is commonly selected for infections caused by Enterobacterales with inducible AmpC β-lactamase production [1] and for severe infections in which *Pseudomonas aeruginosa* is a concern, including febrile neutropenia [2].

As CFPM is predominantly eliminated via the kidneys, dose adjustment based on renal function is routinely performed clinically, most commonly using creatinine clearance (Ccr) [3–5]. CFPM exhibits central nervous system penetration and has been associated with the highest number of reported encephalopathy cases among β-lactam antibiotics [6]. Therefore, CFPM-associated neurotoxicity remains a clinically relevant concern, particularly in patients with impaired renal function [7–9]. Although the reported incidence rates vary, some studies have reported rates approaching 15% [10]. Given that neurological manifestations such as impaired consciousness frequently occur in critically ill patients, CFPM-related neurotoxicity may be under-recognized in routine clinical settings. An exposure–toxicity relationship has been suggested, with an increasing incidence of neurotoxicity at higher trough concentrations [11–14]. Owing to this concern, hesitation regarding the de-escalation from carbapenems to CFPM has occasionally been observed clinically. Therefore, from the perspective of antimicrobial stewardship and antimicrobial resistance control, establishing clinical strategies to minimize the risk of CFPM-associated neurotoxicity is of considerable importance.

Serum creatinine (Scr), the basis of commonly used renal function indices, such as Ccr and creatinine-based estimated glomerular filtration rate (eGFR), is influenced by muscle mass and may not accurately reflect renal function in elderly, low-body-weight, or frail patients. Consequently, reliance solely on Scr-based indices may cause inappropriate dose selection in patients requiring dose reduction [15]. Cystatin C has recently been proposed as a renal function marker independent of muscle mass, and may provide a more reliable estimate of renal function for dose adjustment of renally eliminated drugs [16].

However, presenting cystatin C as a superior marker of renal function is insufficient for individualized dosing strategies to be applied clinically. Rather, the clinical significance of the discrepancies between Scr-based renal function indices and cystatin C-based estimates warrants further clarification. Such discrepancies may lead to differences in the recommended dosing and potentially contribute to excessive CFPM exposure.

In the present study, renal function was evaluated using commonly applied clinical parameters, including Ccr calculated using the Cockcroft–Gault equation, eGFR derived from the Japanese equation based on Scr, and cystatin C-based eGFR (eGFRcys) calculated using the Japanese equation based on cystatin C (104 × cystatin C ^-1.019 × 0.996^Age − 8 for males; multiplied by 0.929 for females) [17]. Particular emphasis was placed on the relationship between these renal function indices and CFPM trough concentrations.

Therefore, identifying the clinical parameters that can predict discrepancies between Scr-based renal function indices and cystatin C-based estimates is essential for optimizing CFPM dose selection and minimizing the risk of neurotoxicity.

In addition to optimizing dose selection, rapid and reliable measurement of plasma CFPM concentrations is essential when neurotoxicity is suspected. Although several analytical methods have been reported [18–22], their applicability under real-world polypharmacy conditions remains unclear. Because CFPM is often co-administered with glycopeptides such as vancomycin (VCM) and teicoplanin (TEIC), as well as clindamycin (CLDM) or metronidazole (MNZ) for anaerobic coverage. However, previous analytical methods have not sufficiently evaluated the chromatographic separation of CFPM from these commonly co-administered agents. Therefore, establishing a selective, stable, and clinically applicable HPLC method that reflects routine clinical practice is warranted.

In this study, we sought to improve the clinical evaluation of CFPM exposure and dose selection by integrating plasma concentration measurements with renal function assessments. Our approach involved developing and validating a practical HPLC assay to quantify CFPM in clinical plasma samples, followed by an evaluation of the clinical significance of discrepancies between Scr-based Ccr and eGFRcys in CFPM dose selection, particularly in relation to elevated trough concentrations, suggesting potential CFPM overexposure. Finally, we aimed to determine whether this discrepancy could be predicted using conventional clinical parameters, thereby providing a safer and more practical framework for individualizing the CFPM doses.

## Methods

### Reagents and standards

Reference standards for CFPM and ceftazidime (CAZ), used as internal standards (IS), were purchased from the Pharmaceutical and Medical Device Regulatory Science Society of Japan (PMRJ; Tokyo, Japan). Liquid chromatography–mass spectrometry (LC–MS)/MS-grade methanol, analytical-grade phosphoric acid, and sodium dihydrogen phosphate were obtained from FUJIFILM Wako Pure Chemical Corporation (Osaka, Japan). Milli-Q water was used for all experiments. Stock solutions of CFPM and CAZ were prepared in water at a concentration of 1 mg/mL and stored at − 80 °C until use. Calibration curves were prepared at six concentrations (2, 5, 10, 50, 100, and 200 µg/mL), and the IS concentration was fixed at 25 µg/mL. CAZ was selected as the IS because of its physicochemical similarity to CFPM and absence of concomitant clinical use, minimizing the risk of chromatographic interference.

### Sample preparation

Plasma samples were pretreated using solid-phase extraction (SPE) with Oasis HLB^®^ 1 cc Vac cartridges (Waters; Milford, MA, USA). The cartridges were conditioned with 1 mL of methanol and equilibrated with 1 mL of water, followed by 1 mL of 50 mM phosphate buffer (pH 2.0), prepared from phosphoric acid and sodium dihydrogen phosphate.

A mixture consisting of 50 µL plasma, 50 µL of IS solution (50 µg/mL), and 300 µL of phosphate buffer (pH 2.0) (total volume 400 µL) was loaded onto the cartridge. After loading, the cartridge was washed with 1 mL phosphate buffer, and the analytes were eluted twice with 0.25 mL methanol (total 0.5 mL). The eluate was evaporated to dryness under nitrogen at 40 °C and reconstituted in 100 µL of solvent corresponding to the initial mobile phase composition. After centrifugation, 90 µL of the supernatant was filtered through a 0.45 μm membrane filter by centrifugation. Finally, 10 µL of the prepared sample was injected into the HPLC system on the same day.

### HPLC conditions

Chromatographic analysis was performed using a SHIMADZU HPLC system equipped with an LC-20AD pump, a CTO-20 A column oven, and an SPD-20AV UV–VIS detector (SHIMADZU; Kyoto, Japan). Separation was achieved using a Shim-pack HPLC packed column (150 mm × 4.6 mm, 5 μm) with a Shim-pack GWS guard column (10 mm × 4.0 mm, 5 μm). The mobile phase consisted of 40 mM phosphate buffer (pH 3.0) as solvent A, and methanol as solvent B. The gradient program was as follows: 0–1 min, 10% B; 1–1.5 min, 15% B; 1.5–10.5 min, 15% B; 10.5–11 min, 10% B; and 11–13 min, 10% B.

The flow rate was set at 1.0 mL/min, the injection volume was 10 µL, and the column temperature was maintained at 35 °C. The detection was performed at a wavelength of 260 nm. The total runtime for each analysis was 13 min.

### Method validation

The method was validated in accordance with the U.S. Food and Drug Administration (FDA) Bioanalytical Method Validation Guidelines for Industry (2018). Linearity and accuracy were evaluated at six concentrations ranging from 2 to 200 µg/mL. Extraction recovery and matrix effects were assessed at 2, 50, 100, and 200 µg/mL by comparing the peak areas of CFPM spiked into blank plasma (L-consera^®^, SHIMADZU) with those of CFPM prepared in water. Intraday precision was evaluated using three replicate measurements on the same day, whereas interday precision was assessed using 12 calibration curves generated over 4 consecutive days. Stability was examined through freeze–thaw testing and long-term storage at − 80 °C. Stability was evaluated by analyzing samples on the day after collection and after storage for 3 6 months at − 80 °C. Freeze–thaw stability was assessed by subjecting the CFPM stock solutions to repeated freeze–thaw cycles. The precision was expressed as the relative standard deviation (RSD, %).

### Study design and patients

This prospective observational study included 22 hospitalized patients who underwent CFPM at Shizuoka General Hospital between August 2024 and February, 2026. The exclusion criteria were age < 18 years, ongoing hemodialysis or peritoneal dialysis, and CFPM treatment duration of ≤ 2 days. Steady state was defined as ≥ 48 h after the initiation of CFPM therapy, and trough blood samples were collected within 10 min before the next scheduled dose. Considering that the elimination half-life of CFPM in healthy subjects is approximately 2 h, this steady-state definition is pharmacokinetically reasonable [23]. Clinical blood samples were collected in 5-mL ethylenediaminetetraacetic acid disodium salt (EDTA-2Na) tubes. After collection, samples were immediately stored at 4 °C and centrifuged within 3 h at 1,500 × g for 10 min at 4 °C. The separated plasma was aliquoted and stored at − 80 °C until analysis. The study protocol was approved by the Ethics Committee of the University of Shizuoka (approval number: 6–26) and the Ethics Committee of Shizuoka General Hospital (approval number: SGHIRB#2024022). This study was supported by a grant from the Japan Research Foundation for Clinical Pharmacology (grant no. 2024J04).

### Outcomes and variables

The objective of this study was to establish a widely applicable and high-precision HPLC method for plasma CFPM quantification and identify factors associated with elevated CFPM trough concentrations under routine Ccr-based dose adjustment. Previous studies have proposed cefepime neurotoxicity-associated trough concentration thresholds ranging from 16 to 49 µg/mL, with no established consensus [14]. Therefore, a trough concentration ≥ 16 µg/mL was adopted as a conservative screening threshold to identify patients with potential cefepime overexposure rather than as a diagnostic threshold for neurotoxicity [11]. Patients were categorized according to the presence or absence of overexposure, and the clinical characteristics were compared between the groups.

Particular emphasis was placed on the relationship between trough concentrations and renal function indices. Renal function indices were calculated using standard equations, including Ccr based on the Cockcroft–Gault equation, eGFR based on the Japanese equation using Scr, and eGFRcys based on the Japanese equation using cystatin C. To account for potential overestimation of renal function in patients with low Scr, Scr values < 0.6 mg/dL were rounded up to 0.6 mg/dL for Ccr calculation, hereafter referred to as Ccr (RU). Furthermore, eGFR and eGFRcys were also evaluated after adjustment for body surface area (BSA), yielding eGFR (BSA) and eGFRcys (BSA), respectively. All renal function parameters were obtained on the same day as the trough sampling. To evaluate the renal function index that best correlated with trough concentrations when used for dose adjustment, correlations were assessed between trough concentrations and the ratio of the total daily dose to each renal function index (daily dose/Ccr, daily dose/Ccr (RU), daily dose/eGFR, daily dose/eGFR (BSA), daily dose/eGFRcys, and daily dose/eGFRcys (BSA)), as previously described [24].

Renal function discrepancy was defined as the ratio of Ccr to eGFRcys (BSA) [Ccr/eGFRcys (BSA)], because Ccr is routinely used for cefepime dose adjustment in clinical practice. The association between Ccr/eGFRcys (BSA) and cefepime concentration was evaluated. Furthermore, factors associated with Ccr/eGFRcys (BSA) were explored to assess whether this discrepancy could be predicted using conventional clinical variables.

Additional variables collected at the time of trough sampling included age, sex, body weight, body mass index (BMI), BSA, aspartate aminotransferase (AST), alanine aminotransferase (ALT), serum albumin (ALB), C-reactive protein (CRP), CFPM single dose, total daily dose, dosing interval, infusion time, time from initial administration to trough sampling, and clinical factors reported to affect cystatin C levels, including malignancy, thyroid dysfunction, corticosteroid use, and cyclosporine use.

### Statistical analysis

Continuous variables were compared between patients with trough concentrations ≥ 16 µg/mL and those with < 16 µg/mL using the Wilcoxon rank-sum test, whereas categorical variables were analyzed using Fisher’s exact test. Associations between CFPM trough concentrations and dose-normalized parameters (daily dose/Ccr, daily dose/rounded Ccr, daily dose/eGFR, daily dose/eGFR (BSA), daily dose/eGFRcys, and daily dose/eGFRcys (BSA)) as well as the Ccr/eGFRcys (BSA) ratio were evaluated using Spearman’s rank correlation coefficient. All statistical tests were two-sided, and a *P* < 0.05 was considered significant. Statistical analyses were performed using the R software (version 4.4.0; R Foundation for Statistical Computing, Vienna, Austria).

## Results

### Method validation

**Fig. 1:**
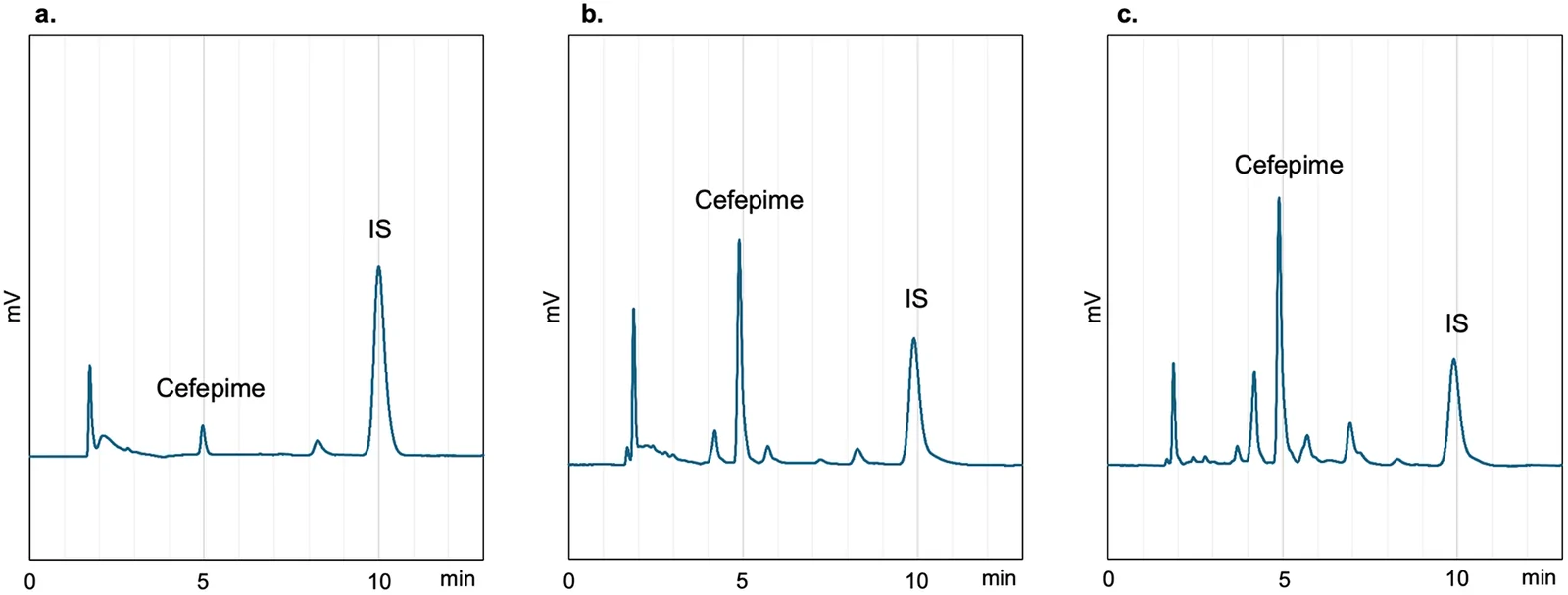
Representative high-performance liquid chromatography chromatograms showing (**a**) blank plasma spiked with cefepime (2 µg/mL) and the internal standard (IS) ceftazidime (25 µg/mL), (**b**) a patient plasma sample obtained during concomitant administration of vancomycin and clindamycin, and (**c**) a patient plasma sample obtained during concomitant administration of teicoplanin and metronidazole. The retention times of cefepime and IS were approximately 4.9 and 9.7 min, respectively

The retention times (Rt) of CFPM and IS were 4.9 and 9.7 min, respectively (Fig. 1).

Evaluation across 50 samples analyzed on different days demonstrated RSD values of 0.57% for CFPM and 1.39% for the IS (Supplemental Digital Content Table S1). Representative chromatograms of plasma spiked with CFPM and the IS, and plasma samples obtained from patients receiving concomitant VCM and CLDM or TEIC and MNZ are shown in Fig. 1. Clear separation of CFPM and the IS was maintained in spiked plasma (Fig. 1a) and in plasma samples obtained from patients receiving VCM, TEIC, CLDM, or MNZ (Fig. 1b and c). Among the 22 patients included in the clinical evaluation, 13 (59.1%) received concomitant treatment with one or more of these antimicrobial agents, and no chromatographic interference was observed in any patient sample. In addition, stability testing using six patient plasma samples demonstrated acceptable CFPM stability after 6 months of storage at − 80 °C, with a mean recovery of 98.3% and an RSD of 5.6%. Furthermore, freeze–thaw stability of stock solutions was confirmed, with consistent concentrations observed after five freeze–thaw cycles for CFPM and four cycles for the IS. The calibration curve shown in Fig. S1 (Supplemental Digital Content) exhibited linearity over the concentration range of 2–200 µg/mL (R² ≥ 0.999).

The intraday (*n* = 3 calibration curves) and interday (*n* = 12 calibration curves over 4 days) reproducibility of regression coefficients was evaluated (Supplemental Digital Content Table S2). A comparison of three regression models—unweighted, 1/X, and 1/X² weighting—revealed that the 1/X²-weighted least-squares model provided the best fit among the tested models. Precision and accuracy were evaluated at concentrations ranging from 2 to 200 µg/mL. The intraday %RSD ranged from 0.43% to 3.50%, whereas the interday %RSD ranged from 4.91% to 15.15%. The accuracy assessed using the back-calculated concentrations remained within ± 3.49% across all concentrations. Accuracy assessed using back-calculated concentrations met the FDA acceptance criteria, remaining within ± 15% across all concentrations and within ± 20% at the LLOQ (2 µg/mL). Accordingly, a 1/X²-weighted regression model was used for sample quantification. The limit of detection (LOD) was 0.5 µg/mL.

Extraction recovery was calculated by comparing the peak areas of post-extracted samples (CFPM spiked into L-Concera^®^ and subjected to SPE) with those of non-extracted standard solutions prepared in water. Mean extraction recoveries were 88.9% at 2 µg/mL, 103.6% at 50 µg/mL, 96.7% at 100 µg/mL, and 110.2% at 200 µg/mL (Supplemental Digital Content Table S3).

Evaluation of matrix effects revealed mean percentage changes of + 7.6% at 2 µg/mL, − 4.5% at 50 µg/mL, − 0.87% at 100 µg/mL, and − 6.46% at 200 µg/mL (Supplemental Digital Content Table S4). All subsequent evaluations of CFPM plasma concentrations were conducted using a validated HPLC method.

### Characteristics of study participants

The baseline characteristics of the 22 enrolled patients are summarized in Table 1.

**Table 1:** Comparison of patient characteristics between those with cefepime trough concentrations ≥ 16 µg/mL and < 16 µg/mL

	All patients (*n* = 22)	CFPM trough concentration		
		< 16 µg/mL (*n* = 10)	≥ 16 µg/mL (*n* = 12)	*P* value
Sex, Male	12 (55%)	7(70%)	5 (42%)	0.23
Age (years)	69 (59, 75)	60 (56, 72)	74 (70, 75)	0.07
Body weight (kg)	53 (46, 66)	58 (51, 68)	51 (45, 63)	0.46
BMI (kg/m²)	22 (19, 25)	21 (18, 23)	23 (20, 26)	0.42
BSA (m^2^)	1.56 (1.41, 1.73)	1.62 (1.55, 1.74)	1.45 (1.40, 1.63)	0.49
Malignancy	8 (36%)	3 (30%)	5 (42%)	0.68
Thyroid dysfunction	1 (5%)	1 (10%)	0 (0%)	0.46
Corticosteroid use	4 (18%)	3 (30%)	1 (8%)	0.29
Cyclosporine use	0 (0%)	0 (0%)	0 (0%)	1.00
Clinical date				
Scr	0.63 (0.55, 0.84)	0.58 (0.49, 0.63)	0.76 (0.56, 1.27)	0.14
Ccr	86 (56, 105)	102 (90, 109)	57 (36, 84)	0.003
Ccr (RU)	77.8 (51.3, 89.8)	89.6 (84.1, 94.8)	51.6 (36.3, 72.8)	< 0.001
eGFR	84 (59, 107)	99 (85, 112)	66 (42, 86)	0.025
eGFR (BSA)	78.9 (51.8, 94.8)	91.2 (86.9, 99.9)	54.0 (37.8, 76.3)	0.011
Cystatin C	0.99 (0.86, 1.68)	0.85 (0.84, 0.92)	1.68 (1.25, 3.00)	< 0.001
eGFRcys	64.5 (35.0, 81.8)	84.5 (76.5, 88.8)	37.0 (18.3, 53.5)	< 0.001
eGFRcys (BSA)	56.6 (27.4, 73.5)	77.7 (67.6, 81.7)	29.6 (17.9, 51.7)	< 0.001
Ccr/eGFRcys (BSA)	1.54 (1.30, 1.85)	1.13 (1.21, 1.44)	1.84 (1.68, 2.20)	0.002
AST	20 (16, 43)	28 (16, 48)	19 (16, 30)	0.40
ALT	25 (10, 35)	33 (11, 38)	19 (10, 26)	0.40
ALB	2.30 (2.10, 2.58)	2.70 (2.30, 3.10)	2.10 (1.98, 2.30)	0.008
CRP	6.1 (3.3, 10.6)	5.9 (3.9, 9.8)	7.0 (3.1, 13.6)	0.76
Dosage regimen				
Single dose (mg)	1000 (1000, 2000)	1000 (1000, 1750)	1000 (1000, 2000)	1.00
Daily dose (mg)	3000 (3000, 4000)	4000 (3000, 3750)	3000 (3000, 6000)	0.82
Dosing interval (h)	8 (8, 8)	8 (8, 8)	8 (8, 8)	0.77
Infusion time (h)	1.00 (1.00, 1.00)	1.00 (1.00, 1.00)	1.00 (1.00, 1.25)	0.12
Time to trough (h)	80 (71, 110)	75 (68, 101)	85 (73, 112)	0.38
Treatment duration (days)	8 (5, 18)	10 (8, 22)	7 (5, 15)	0.35
Trough (µg/mL)	17.4 (7.7, 25.8)	7.0 (3.8, 8.5)	25.3 (20.7, 37.0)	< 0.001

Data were presented as frequencies (percentages) or medians (interquartile ranges)

ALB, serum albumin; ALT, alanine aminotransferase; AST, aspartate aminotransferase; BMI, body mass index; BSA, body surface area; Ccr, creatinine clearance; Ccr(RU), creatinine clearance calculated using a rounded serum creatinine value of 0.6 mg/dL; CRP, C-reactive protein; eGFR, estimated glomerular filtration rate; Scr, serum creatinine

Twelve patients (55%) exhibited trough concentrations ≥ 16 µg/mL, meeting the predefined criterion for overexposure. Patients with trough concentrations ≥ 16 µg/mL had significantly lower values for all renal function indices, including Ccr, Ccr (RU), eGFR, eGFR (BSA), eGFRcys, and eGFRcys (BSA), as well as higher cystatin C levels and lower serum albumin levels, compared with those with trough concentrations < 16 µg/mL. In addition, the Ccr/eGFRcys (BSA) ratio was significantly higher in the overexposure group.

The CFPM single dose, total daily dose, dosing interval, infusion duration, and time from the initiation of therapy to trough sampling did not differ significantly between the groups. The liver function and inflammatory markers (AST and ALT activities, and CRP level) were comparable. In addition, the prevalence of malignancy, thyroid dysfunction, corticosteroid use, and cyclosporine use did not differ significantly between the groups. No patient developed clinically evident neurotoxicity during the study period.

### Association between trough concentrations and renal function indices

**Fig. 2:**
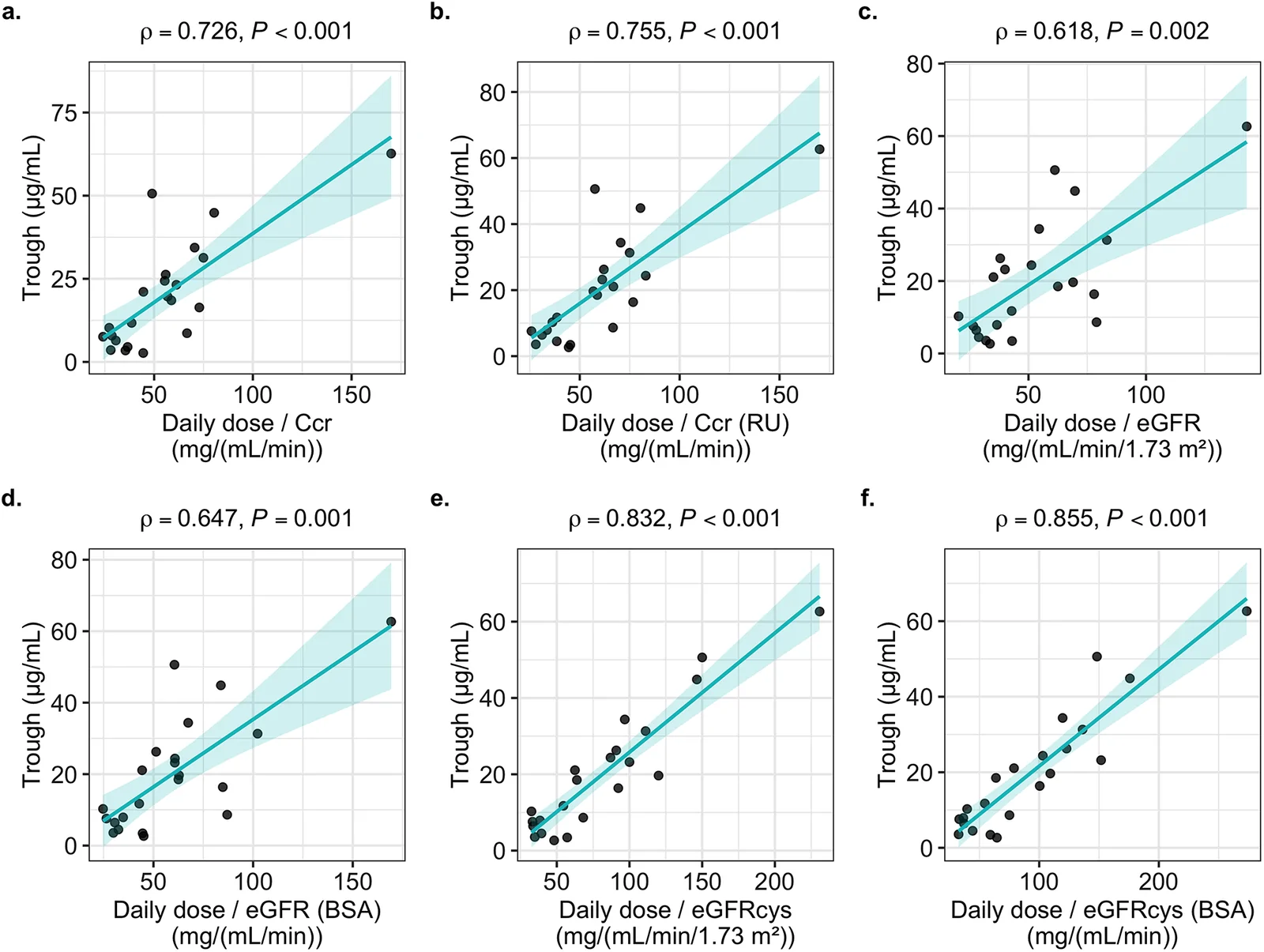
Spearman’s rank correlation analyses between cefepime trough concentrations and the renal function-adjusted daily dose (daily dose divided by each renal function index). (**a**) Creatinine clearance (Ccr), (**b**) creatinine clearance calculated using the rounding-up method with serum creatinine values < 0.6 mg/dL adjusted to 0.6 mg/dL (Ccr [RU]), (**c**) estimated glomerular filtration rate (eGFR), (**d**) body surface area-adjusted eGFR (eGFR [BSA]), (**e**) cystatin C-based eGFR (eGFRcys), and (**f**) body surface area-adjusted cystatin C-based eGFR (eGFRcys [BSA])

The relationships between trough concentrations and daily doses normalized to renal function indices (daily dose/renal function parameters) are shown in Fig. 2.

Panels a–f in Fig. 2 illustrate the associations between trough concentration and normalized daily dose calculated using different renal function indices: Ccr (a), Ccr (RU) (b), eGFR (c), BSA-adjusted eGFR (d), eGFRcys (e) and BSA-adjusted eGFRcys (BSA) (f).

Spearman’s rank correlation analysis revealed significant positive correlations between the normalized dose and trough concentration for all evaluated renal function indices. The correlation coefficients (ρ) and corresponding *P*-values were as follows: Ccr (ρ = 0.73, *P* < 0.001), Ccr calculated using the RU method (ρ = 0.76, *P* < 0.001), eGFR (ρ = 0.62, *P* = 0.002), BSA-adjusted eGFR (ρ = 0.65, *P* = 0.001), eGFRcys (ρ = 0.83, *P* < 0.001) and BSA-adjusted eGFRcys (ρ = 0.86, *P* < 0.001). Among these indices, normalization using eGFRcys (BSA) (Fig. 2f) showed the strongest correlation with the trough concentration.

### Association between trough concentrations and Ccr/eGFRcys (BSA)

As shown in Fig. 2f, the highest correlation was observed between CFPM trough concentrations and the daily dose normalized to eGFRcys (BSA). A higher Ccr/eGFRcys (BSA) ratio was significantly associated with trough concentrations ≥ 16 µg/mL in Table 1 (1.84 [1.68–2.20] vs. 1.13 [1.21–1.44], *P* = 0.002).

**Fig. 3:**
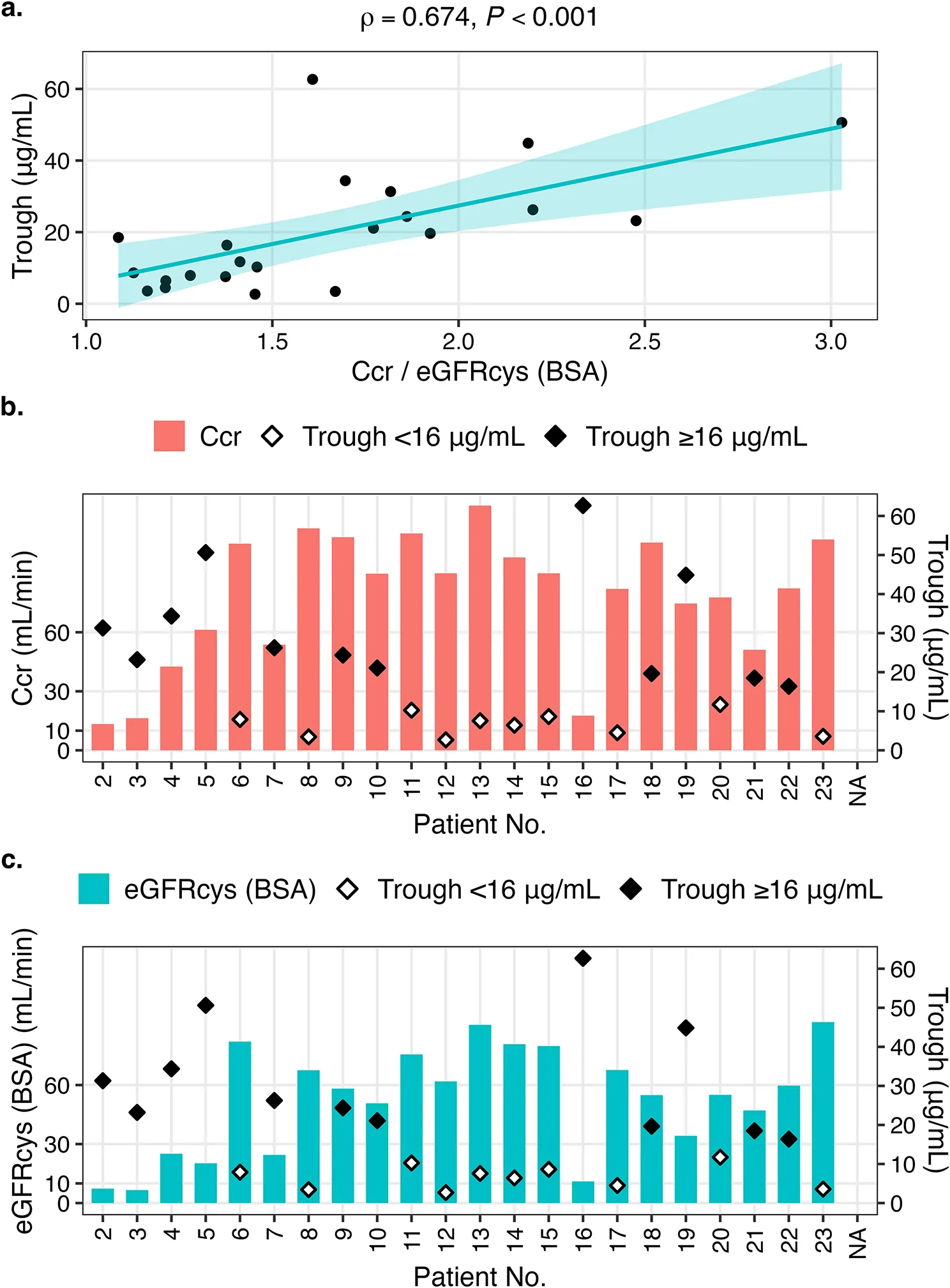
Relationships between renal function indices and cefepime trough concentrations. (**a**) Spearman’s rank correlation between cefepime trough concentrations and the Ccr/eGFRcys (BSA) ratio, representing the discrepancy between creatinine clearance (Ccr) and body surface area-adjusted cystatin C-based estimated glomerular filtration rate (eGFRcys [BSA]). (**b**) Relationship between Ccr and cefepime trough concentrations in individual patients (*n* = 22). (**c**) Relationship between eGFRcys (BSA) and cefepime trough concentrations in the same patients. In panels (**b**) and (**c**), patients are arranged from left to right according to patient number (recruitment order). Open diamonds indicate trough concentrations < 16 µg/mL, and filled diamonds indicate trough concentrations ≥ 16 µg/mL. Tick labels denote renal function categories used for cefepime dose adjustment (< 10, 10–30, 30–60, and ≥ 60 mL/min)

To investigate the effect of renal function parameters of Ccr/eGFRcys (BSA) on CFPM trough concentrations, the correlation between Ccr/eGFRcys (BSA) and CFPM trough concentrations, and the relationship between Ccr or eGFRcys (BSA) and each CFPM trough concentration were evaluated. The results are shown in Fig. 3.

Trough concentrations were significantly and positively correlated with the Ccr/eGFRcys (BSA) ratio (ρ = 0.67, *P* < 0.001) (Fig. 3a). Because this ratio reflects the discrepancy between creatinine-based and cystatin C-based renal function estimates, the relationships between renal function indices and trough concentrations were further examined at the individual patient level. Figure 3b shows the relationship between Ccr and trough concentrations, whereas Fig. 3c depicts the relationship between eGFRcys (BSA) and trough concentrations. In most patients with preserved renal function based on Ccr (≥ 60 mL/min), trough concentrations were generally maintained below 16 µg/mL.

However, a comparison of Fig. 3b and c revealed that several patients (patient no. 5, 9, 10, 18, 19 and 22) exhibited trough concentrations ≥ 16 µg/mL despite Ccr values suggesting preserved renal function. In these specific cases, eGFRcys (BSA) levels were either below or near the threshold of 60 mL/min. Moreover, all six patients with impaired renal function defined by Ccr (< 60 mL/min) had trough concentrations ≥ 16 µg/mL.

**Fig. 4:**
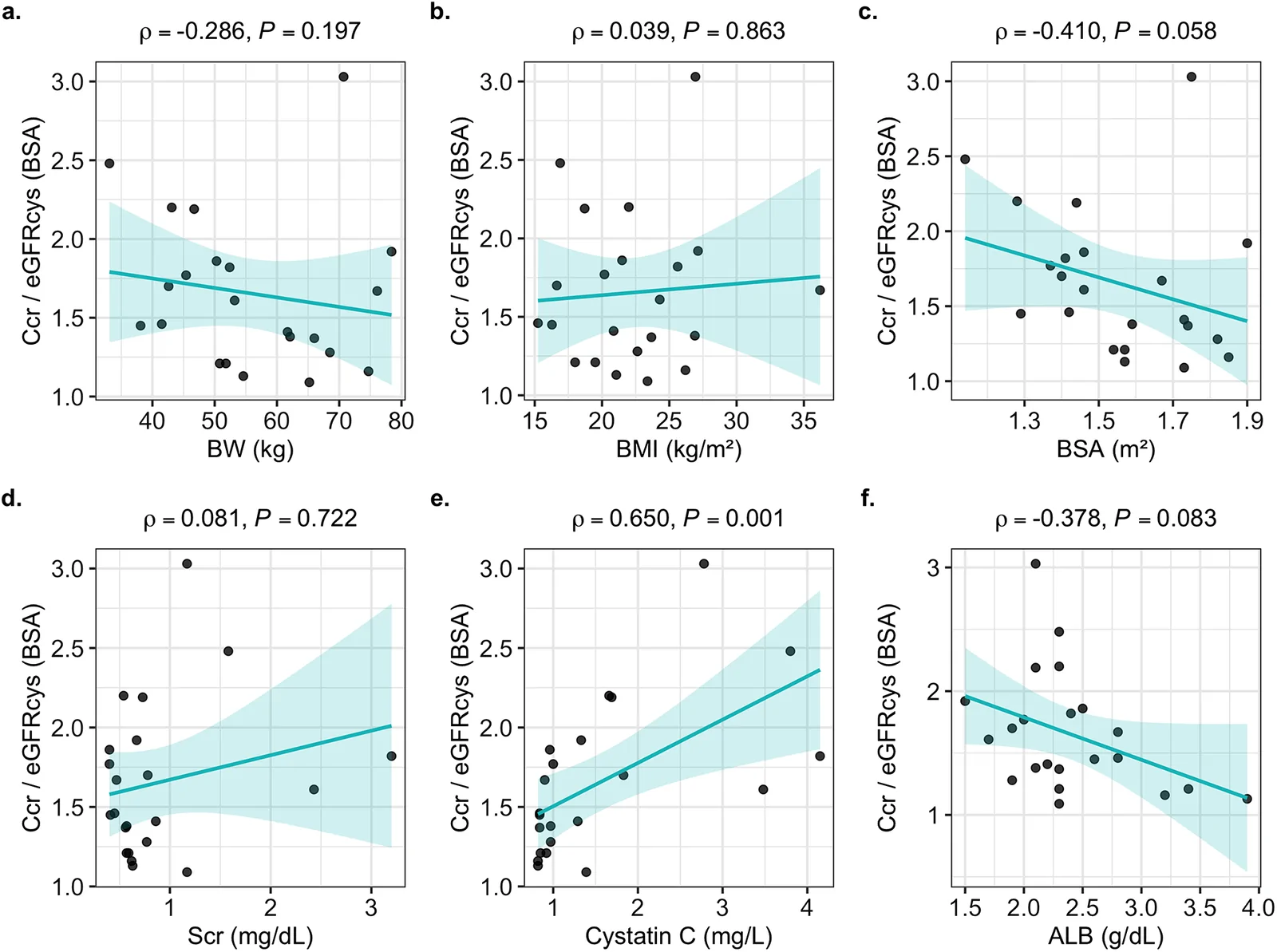
Spearman’s rank correlation analyses between the ratio of creatinine clearance to body surface area-adjusted cystatin C-based estimated glomerular filtration rate (Ccr/eGFRcys [BSA]) and the following variables: (**a**) body weight (BW), (**b**) body mass index (BMI), (**c**) body surface area (BSA), (**d**) serum creatinine (Scr), (**e**) cystatin C, and (**f**) serum albumin (ALB)

Correlations between Ccr/eGFRcys (BSA) and body weight (a), BMI (b), BSA (c), Scr (d), cystatin C (e), and ALB (f) were evaluated to explore potential factors contributing to the discordance between Ccr and eGFRcys (BSA) (Fig. 4). Among these variables, Ccr/eGFRcys (BSA) showed the strongest positive correlation with cystatin C (ρ = 0.65, *P* = 0.001), whereas Scr and body composition-related parameters showed no significant associations.

## Discussion

In this study, we established a conventional HPLC-based method for quantifying plasma CFPM concentrations and confirmed its analytical validity and practical applicability in routine clinical settings. An important objective of this study was to develop a reliable and widely implemented assay that can support concentration-guided antimicrobial therapy without requiring LC–MS/MS. This method demonstrated excellent linearity, precision, and recovery with minimal matrix effects. A major objective of this study was to establish an HPLC method applicable to real-world polypharmacy conditions. Because CFPM is frequently co-administered with glycopeptides and anti-anaerobic agents, analytical interference from concomitant medications may compromise assay performance. Consistent with this objective, CFPM and the IS were clearly separated from co-administered antimicrobial agents, including VCM, TEIC, MNZ, and CLDM, in clinical plasma samples (Fig. 1). Notably, 59.1% of the patients (13/22) included in the clinical evaluation received one or more of these antimicrobial agents, and no chromatographic interference was observed. These results indicate that the present assay overcomes an important limitation of previously reported methods and provides a practical approach for routine therapeutic monitoring of CFPM.

From the results shown in Tables 1 and 55% of patients exhibited trough concentrations ≥ 16 µg/mL, a level previously reported to be associated with an increased risk of neurotoxicity [11]. Notably, these elevated trough concentrations were observed despite routine dose adjustments based on Ccr. This suggests that conventional Ccr-guided dosing may not sufficiently prevent CFPM overexposure in real-world clinical practice. Importantly, no significant differences were observed in CFPM dose, dosing interval, or infusion duration between patients with and without overexposure, indicating that elevated trough concentrations were unlikely to be attributable to excessive prescribed dosing, but rather to differences in renal function estimation. Because the overexposure group included a larger proportion of patients with impaired renal function, the possibility that trough concentrations were measured before steady state was considered. Although cefepime elimination can be prolonged in patients with severe renal impairment, with a reported half-life of up to 19 h [14], no patient in this study had a Ccr < 10 mL/min. Moreover, trough samples in the overexposure group were collected a median of 85 h (interquartile range, 73–112 h) after treatment initiation, suggesting that the influence of sampling before steady state was likely minimal.

Among the evaluated renal function indices, eGFRcys (BSA) demonstrated the strongest correlation with trough concentration, outperforming both Ccr- and creatinine-based eGFR. Furthermore, eGFRcys (BSA) values were consistently lower than the Ccr values in all patients included in this study (Fig. 3). As shown in Table 1, no significant differences were observed between the low- (< 16 µg/mL) and high-trough (≥ 16 µg/mL) groups in the prevalence of malignancy, thyroid dysfunction, corticosteroid use, or cyclosporine use, all of which have been reported to influence cystatin C levels. Therefore, the stronger association between eGFRcys (BSA) and CFPM trough concentrations is unlikely to be explained by an imbalance in these potential confounding factors. Notably, in patients with preserved renal function according to eGFRcys (BSA) (eGFRcys ≥ 60), the risk of excessive exposure or neurotoxicity under standard dosing appeared to be low. In fact, among the 9 patients with eGFRcys (BSA) ≥ 60, all patients (100%) maintained trough concentrations below 16 µg/mL.

In addition, when dosing decisions are based on eGFRcys (BSA), dose reduction may be recommended more frequently than in conventional Ccr-based dosing, potentially raising concerns regarding underdosing. However, our findings suggest that these risks are not substantial. Among patients with Ccr ≥ 60 but eGFRcys (BSA) < 60, all six patients (100%) exhibited trough concentrations ≥ 16 µg/mL. These findings suggest that dose adjustment based solely on Ccr may overestimate renal function, resulting in excessive CFPM exposure, and highlight the potential clinical value of incorporating eGFRcys (BSA) into dosing considerations.

Importantly, the discrepancy between Ccr and eGFRcys (BSA) levels was significantly associated with elevated trough concentrations (Fig. 3). In addition, an analysis of factors related to this discrepancy revealed that the Ccr/eGFRcys (BSA) ratio was significantly correlated with serum cystatin C, but not with Scr or body weight (Fig. 4). These findings support broader implementation of cystatin C measurement in routine clinical practice, including in patients with apparently preserved renal function based on serum creatinine, as selective testing based solely on low Scr levels or suspected reduced muscle mass may overlook clinically relevant discrepancies between Ccr and eGFRcys (BSA).

In contrast, among patients with Ccr < 60 mL/min, trough concentrations were consistently ≥ 16 µg/mL, regardless of the magnitude of discrepancy between Ccr and eGFRcys (BSA). This observation suggests that in patients with impaired renal function, the prediction of CFPM exposure based solely on renal function indices, including eGFRcys (BSA), may not always be sufficient.

Although cystatin C-based renal assessment has been reported to improve the prediction of CFPM exposure compared with creatinine-based indices [25], a similar tendency was observed in the present study. However, routine measurement of cystatin C may not always be feasible because of limitations related to reimbursement policies, assay availability, and institutional practice. Therefore, Ccr remains a widely available and clinically useful screening tool in daily practice.

Furthermore, the present findings further suggest that reliance on renal function markers alone, even when using cystatin C-based estimates, may be inadequate in patients with reduced renal function. In such patients, direct measurement of plasma concentrations and subsequent dose adjustment based on observed values may represent the most reliable strategy for preventing excessive exposure.

In the present study, a substantial proportion of patients exceeded the proposed neurotoxicity-associated threshold of 16 µg/mL; however, no clinically evident neurotoxicity was observed. Notably, several patients had trough concentrations exceeding 40 µg/mL, yet none developed neurotoxicity. Our findings suggest that cefepime trough concentration alone is insufficient to accurately predict the development of neurotoxicity. Because the reported median time to onset of cefepime-associated neurotoxicity is approximately 4 days [7], and the median treatment duration in our cohort was 8 days, the absence of neurotoxicity is unlikely to be explained by insufficient treatment duration. Rather, the plasma concentration alone may not fully determine the risk of neurotoxicity. Although systemic exposure remains an important determinant, it should be interpreted in a broader pathophysiological context. Differences in blood–brain barrier permeability and cerebrospinal fluid penetration may result in variable central nervous system exposure, implying that plasma trough concentrations alone may not fully explain cefepime-associated neurotoxicity [26]. In addition, β-lactam antibiotics, including cefepime, have been suggested to exert neurotoxic effects through inhibition of γ-aminobutyric acid (GABA)-A receptors, which may further contribute to interindividual susceptibility [27, 28].

This study had several limitations. First, this was a single-center study with a limited sample size, which may have restricted the generalizability of our findings. Second, no cases of clinically apparent neurotoxicity were observed in this cohort, precluding the direct evaluation of exposure–toxicity relationships. Third, although eGFRcys (BSA) appeared to be a useful marker for predicting cefepime exposure, its utility for guiding dose optimization was not directly evaluated in this study and should be confirmed in future prospective studies. Finally, because this study was conducted in routine clinical practice as an observational study, causal inferences regarding optimal dosing strategies remain limited.

Despite these limitations, the consistent association between elevated trough concentrations and the discordance between Ccr and eGFRcys (BSA) provides clinically meaningful insights. The integration of cystatin C-based renal assessments with reliable concentration measurements may represent a rational strategy to minimize CFPM overexposure while preserving antimicrobial efficacy. The validated HPLC method established in this study provides a practical analytical foundation for such clinical implementation and may facilitate rapid concentration assessment when neurotoxicity is suspected. Therefore, although a cystatin C-based assessment may improve the prediction of exposure in patients with preserved renal function, patients with moderate-to-severe renal impairment may require direct concentration monitoring. In this context, TDM-supported dosing may represent an important approach for individualized therapy and reduction of neurotoxicity risk.

## Conclusions

A conventional HPLC method for plasma cefepime quantification was successfully established, and it demonstrated reliable performance under clinical conditions. Ccr-guided dosing frequently resulted in trough concentrations ≥ 16 µg/mL, whereas eGFRcys (BSA) showed a stronger association with CFPM exposure than Ccr, suggesting that it may be a useful marker for predicting CFPM exposure. Notably, 55% of patients exceeded the proposed neurotoxicity-associated threshold of 16 µg/mL, suggesting a potentially substantial risk of cefepime overexposure in routine clinical practice. However, in patients with impaired renal function, prediction of exposure based solely on renal function indices may be insufficient. Under such conditions, direct concentration monitoring using this practical HPLC assay may facilitate individualized dose optimization and improve the detection of cefepime overexposure.

## Supplementary Information

Below is the link to the electronic supplementary material.

Supplementary Material 1

## Data Availability

All data generated or analyzed during this study are included in this published article.

## Abbreviations

ALB: Serum albumin
ALT: Alanine aminotransferase
AST: Aspartate aminotransferase
BMI: Body mass index
BSA: Body surface area
CAZ: Ceftazidime
Ccr: Creatinine clearance
CFPM: Cefepime
CLDM: Clindamycin
CRP: C-reactive protein
eGFR: estimated glomerular filtration rate
eGFR(BSA): Body surface area-adjusted estimated glomerular filtration rate
eGFRcys: Cystatin C-based estimated glomerular filtration rate
eGFRcys(BSA): Body surface area-adjusted cystatin C-based estimated glomerular filtration rate
GABA: γ-aminobutyric acid
HPLC: High-performance liquid chromatography
IS: Internal standard
LC–MS/MS: Liquid chromatography–tandem mass spectrometry
LLOQ: Lower limit of quantification
LOD: Limit of detection
MNZ: Metronidazole
RSD: Relative standard deviation
RU: Rounding-up
Scr: Serum creatinine
SPE: Solid-phase extraction
TEIC: Teicoplanin
TDM: Therapeutic drug monitoring
VCM: Vancomycin

## References

[1] Tamma PD, Girdwood SCT, Gopaul R, et al. The use of cefepime for treating AmpC β-lactamase-producing enterobacteriaceae. Clin Infect Dis. 2013;57:781–8. 10.1093/cid/cit395. 23759352

[2] Freifeld AG, Bow EJ, Sepkowitz KA, et al. Clinical practice guideline for the use of antimicrobial agents in neutropenic patients with cancer: 2010 Update by the Infectious Diseases Society of America. Clin Infect Dis. 2011;52:e56–93. 10.1093/cid/cir073. 21258094

[3] Kuti JL, Nicolau DP. Optimal cefepime and meropenem dosing for ventilator-associated pneumonia patients with reduced renal function: An update to our clinical pathway. J Crit Care. 2010;25:155–6. 10.1016/j.jcrc.2010.01.001. 20189057

[4] Lodise TP, Lomaestro BM, Drusano GL. Application of antimicrobial pharmacodynamic concepts into clinical practice: Focus on β-lactam antibiotics - Insights from the Society of Infectious Diseases Pharmacists. Pharmacotherapy. 2006;26:1320–32. 10.1592/phco.26.9.1320. 16945055

[5] Tam VH, McKinnon PS, Akins RL, et al. Pharmacokinetics and pharmacodynamics of cefepime in patients with various degrees of renal function. Antimicrob Agents Chemother. 2003;47:1853–61. 10.1128/AAC.47.6.1853-1861.2003. PMC155813 12760858

[6] Lacroix C, Kheloufi F, Montastruc F, et al. Serious central nervous system side effects of cephalosporins: A national analysis of serious reports registered in the French Pharmacovigilance Database. J Neurol Sci. 2019;398:196–201. 10.1016/j.jns.2019.01.018. 30683462

[7] Payne LE, Gagnon DJ, Riker RR, et al. Cefepime-induced neurotoxicity: A systematic review. Crit Care. 2017;21:276. 10.1186/s13054-017-1856-1. PMC5686900 29137682

[8] Jeon JY, Cho YW, Moon HJ. Cefepime-induced encephalopathy in a tertiary medical center in Korea. J Clin Neurol. 2020;16:408–15. 10.3988/jcn.2020.16.3.408. PMC7354975 32657061

[9] Li HT, Lee CH, Wu T, et al. Clinical, Electroencephalographic Features and Prognostic Factors of Cefepime-Induced Neurotoxicity: A Retrospective Study. Neurocrit Care. 2019;31:329–37. 10.1007/s12028-019-00682-y. 30756319

[10] Fugate JE, Kalimullah EA, Hocker SE, et al. Cefepime neurotoxicity in the intensive care unit: a cause of severe, underappreciated encephalopathy. Crit Care. 2013;17:R264. 10.1186/cc13094. PMC4057506 24200036

[11] Boschung-Pasquier L, Atkinson A, Kastner LK, et al. Cefepime neurotoxicity: thresholds and risk factors. A retrospective cohort study. Clin Microbiol Infect. 2020;26:333–9. 10.1016/j.cmi.2019.06.028. 31284030

[12] Singh TD, O’Horo JC, Day CN, et al. Cefepime is Associated with Acute Encephalopathy in Critically Ill Patients: A Retrospective Case–Control Study. Neurocrit Care. 2020;33:695–700. 10.1007/s12028-020-01035-w. PMC7329265 32613426

[13] Muradbegovic J, Leidi A, Serratrice J. Cefepime-induced encephalopathy. Rev Med Suisse. 2019;15:2210–2. 31778052

[14] Pais GM, Chang J, Barreto EF, et al. Clinical Pharmacokinetics and Pharmacodynamics of Cefepime. Clin Pharmacokinet. 2022;61:929–53. 10.1007/s40262-022-01137-y. PMC9345683 35764774

[15] Hanna PE, Ouyang T, Tahir I, et al. Sarcopenia, adiposity and large discordance between cystatin C and creatinine-based estimated glomerular filtration rate in patients with cancer. J Cachexia Sarcopenia Muscle. 2024;15:1187–98. 10.1002/jcsm.13469. PMC11154767 38646842

[16] Brou NA, Jacqz-Aigrain E, Zhao W. Cystatin C as a potential biomarker for dosing of renally excreted drugs. Br J Clin Pharmacol. 2015;80:20–7. 10.1111/bcp.12602. PMC4500321 25655191

[17] Horio M, Imai E, Yasuda Y, et al. Collaborators Developing the Japanese Equation for Estimated GFR. GFR estimation using standardized serum cystatin C in Japan. Am J Kidney Dis. 2013;61:197–203. 10.1053/j.ajkd.2012.07.007. 22892396

[18] Calahorra B, Campanero MA, Sádaba B, et al. Rapid high-performance liquid chromatographic determination of cefepime in human plasma. Biomed Chromatogr. 1999;13:272–5. 10.1002/(SICI)1099-0801(199906)13:4<272::AID-BMC842>3.0.CO;2-0. 10416059

[19] Kalyani L, Rao CVN. Stability indicating RP-HPLC method development and validation of cefepime and amikacin in pure and pharmaceutical dosage forms. Braz J Pharm Sci. 2018;54. 10.1590/s2175-97902018000317258.

[20] Tamboli SR, Patil DD. RP-HPLC method for simultaneous estimation of cefepime hydrochloride and tazobactam sodium in bulk and pharmaceuticals. J Chem. 2013;208057. 10.1155/2013/208057.

[21] Valassis IN, Parissi-Poulou M, Macheras P. Quantitative Determination of Cefepime in Plasma and Vitreous Fluid by High-Performance Liquid Chromatography. J Chromatogr B Biomed Sci Appl. 1999;721:249–55. 10.1016/s0378-4347(98)00468-x. 10052697

[22] Nemutlu E, Kir S, Katlan D, Beksaç MS. Simultaneous multiresponse optimization of an HPLC method to separate seven cephalosporins in plasma and amniotic fluid: Application to validation and quantification of cefepime, cefixime and cefoperazone. Talanta. 2009;80:117–26. 10.1016/j.talanta.2009.06.034. 19782200

[23] Barbhaiya RH, Forgue ST, Gleason CR, et al. Pharmacokinetics of cefepime after single and multiple intravenous administrations in healthy subjects. Antimicrob Agents Chemother. 1992;36:552–7. 10.1128/AAC.36.3.552. PMC190556 1622165

[24] Lamoth F, Buclin T, Pascual A, et al. High cefepime plasma concentrations and neurological toxicity in febrile neutropenic patients with mild impairment of renal function. Antimicrob Agents Chemother. 2010;54:4360–7. 10.1128/AAC.01595-08. PMC2944571 20625153

[25] Kim MC, Kim SO, Kim SH, et al. Efficacy and safety of cystatin C-guided renal dose adjustment of cefepime treatment in hospitalized patients with Pneumonia. J Clin Med. 2020;9:1–12. 10.3390/jcm9092803. PMC7564664 32872649

[26] Durand-Maugard C, Lemaire-Hurtel AS, Gras-Champel V, et al. Blood and CSF monitoring of cefepime-induced neurotoxicity: Nine case reports. J Antimicrob Chemother. 2012;67:1297–9. 10.1093/jac/dks012. 22298349

[27] Amakhin DV, Soboleva EB, Zaitsev AV. Cephalosporin antibiotics are weak blockers of GABAa receptor-mediated synaptic transmission in rat brain slices. Biochem Biophys Res Commun. 2018;499:868–74. 10.1016/j.bbrc.2018.04.008. 29625107

[28] Sugimoto M, Uchida I, Mashimo T, et al. Evidence for the involvement of GABAA receptor blockade in convulsions induced by cephalosporins. Neuropharmacology. 2003;45:304–14. 10.1016/s0028-3908(03)00188-6. 12871648

