# Supplementary material for: Clinical applicability of cystatin C-based renal function assessment and HPLC-based therapeutic drug monitoring for predicting cefepime overexposure
Source: J Pharm Health Care Sci. 2026 Aug 13;12:97. doi: 10.1186/s40780-026-00619-6 (PMC13749964; doi:10.1186/s40780-026-00619-6)
Supplement: Supplementary file 1 [file 40780_2026_619_MOESM1_ESM.docx]

**Supplemental Digital Content**

**Table S1.** Retention times (Rt) of cefepime and the internal standard ceftazidime in the HPLC method

|  | Mean Rt (min) | SD (min) | %RSD | n |
| --- | --- | --- | --- | --- |
| Cefepime | 4.92 | 0.03 | 0.57 | 50 |
| Ceftazidime (IS) | 9.74 | 0.14 | 1.39 | 50 |

**Table S2.** Intra- and inter-day precision and accuracy of cefepime quantification by HPLC using an internal standard method with 1/X² weighted calibration

| Conc (µg/mL) | Cefepime / IS | | | | | | |
| --- | --- | --- | --- | --- | --- | --- | --- |
|  | Intra-day (n=3) | | | | Inter-day (n=12) | | |
|  | %RSD | %Accuracy | | | %RSD | | %Accuracy |
| 2 | 2.68 | 2.19 | | | 15.15 | | 2.42 |
| 5 | 2.90 | 2.72 | | | 13.95 | | 3.25 |
| 10 | 3.50 | | 2.83 | 12.70 | | 3.49 | |
| 50 | 1.54 | | 1.52 | 8.92 | | 1.30 | |
| 100 | 0.43 | | 0.38 | 6.87 | | 1.28 | |
| 200 | 1.56 | | 1.42 | 4.91 | | 3.18 | |

**Table S3**. Extraction recovery of cefepime using the post-extraction addition method

| Conc (μg/mL) | Post-extracted Area Ratio (Mean) | Extraction Recovery (%) | Mean (%) |
| --- | --- | --- | --- |
| 2 | 0.06 | 87.61 / 90.24 | 88.93 |
| 50 | 0.87 | 103.04 / 104.07 | 103.55 |
| 100 | 1.81 | 100.35 / 92.97 | 96.66 |
| 200 | 3.29 | 121.22 / 99.15 | 110.19 |

**Table S4**. Matrix Effect of cefepime using the post-extraction addition method

| Conc (μg/mL) | Plasma-dissolved Area Ratio (Mean) | Matrix Effect (%) | Mean (%) |
| --- | --- | --- | --- |
| 2 | 0.05 | 6.66 / 8.54 | 7.60 |
| 50 | 0.83 | −3.67 / −5.33 | −4.50 |
| 100 | 1.79 | −1.01 / −0.73 | −0.87 |
| 200 | 3.39 | −6.42 / −6.50 | −6.46 |

**Fig. S1**
